# Different approaches to assess extracellular vesicles from bovine colostrum

**DOI:** 10.3168/jdsc.2025-0907

**Published:** 2026-02-13

**Authors:** Rafaela R. Santos, Audrey Brooks, Luciana M. Kluppel, Brian Haldeman, Ulrich Bickel, Fernanda Rosa

**Affiliations:** 1 School of Veterinary Medicine, Texas Tech University, Amarillo, TX 79106; 2 Unchained Labs, Pleasanton, CA 94588; 3 Jerry H. Hodges School of Pharmacy, Texas Tech University Health Sciences Center, Amarillo, TX 79106; 4 Center for Blood-Brain Barrier Research, Texas Tech University Health Sciences Center, Amarillo, TX 79106

## Abstract

Extracellular vesicles (EV) are lipid bilayer nanoparticles secreted by most cell types and implicated in intercellular communication, immune regulation, and disease processes. Milk-derived EV, particularly those present in bovine colostrum (BCEV), may play critical roles in neonatal calf development and immunity; however, their characterization remains limited due to methodological challenges in isolating pure vesicle populations. This study aimed to evaluate different methods to detect and characterize BCEV. We isolated BCEV from colostrum of multiparous dairy cows using differential ultracentrifugation and employed complementary analytical techniques to evaluate their morphology, size distribution, and EV-associated markers. Dynamic light scattering indicated an average BCEV diameter of 127.3 ± 9.5 nm, whereas single-particle interferometric reflectance imaging sensing revealed a smaller population of 40- to 45-nm vesicles expressing CD9 and CD63. Transmission electron microscopy further confirmed spherical vesicles averaging 38.7 ± 2 nm, whereas cryogenic electron microscopy visualized intact lipid bilayer structures. These findings demonstrate that BCEV comprise heterogeneous populations detectable by multiple complementary approaches and underscore the importance of combined methodologies for their accurate characterization.

## Highlights

• Bovine colostrum-derived extracellular vesicles comprise heterogeneous vesicle populations.
• Complementary methods are required for accurate BCEV detection.
• Small EV expressing CD9 and CD63 are reliably identified through combined approaches.

## Graphical Abstract

**Figure:**
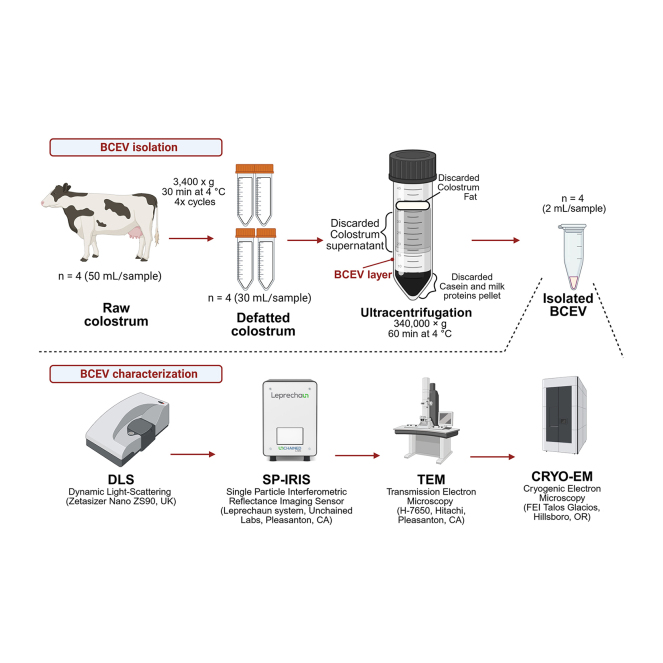
**Summary:** Bovine colostrum-derived extracellular vesicles (BCEV) were isolated from defatted colostrum from Holstein dairy cows (n = 4) via a differential ultracentrifugation method. Characterization of BCEV was performed using dynamic light scattering (DLS), single-particle interferometric reflectance imaging sensing (SP-IRIS), transmission electron microscopy (TEM), and cryogenic electron microscopy (cryo-EM). Combining imaging with quantitative, marker-based detection (e.g., SP-IRIS) results in a more robust identification and characterization of extracellular vesicles (EV) in bovine colostrum. Graphical abstract created with BioRender.com (https://BioRender.com/4fhi7ry).

Extracellular vesicles (**EV**) are lipid bilayer molecules ranging from 30 to 200 nm (small EV) or >200 nm in diameter (large EV) that can be isolated from several biological fluids, including blood, urine, lymph, cerebrospinal fluid, saliva, and milk (Théry et al., 2002, Welsh et al., 2024). Extracellular vesicle biogenesis occurs through 3 main mechanisms: release by merging of multivesicular bodies with the cytoplasmic membrane; protrusion of the cytoplasmic membrane; and apoptosis-associated vesicle formation, in which programmed cell death drives the release of apoptotic bodies in the form of larger cell vesicles carrying cellular debris and cytoplasmic and nuclear components (Maas et al., 2017). Extracellular vesicles carry proteins, lipids, DNA, and different types of RNAs, including mRNA, long noncoding RNAs, and small noncoding RNAs such as microRNAs that can be transferred to recipient cells by endocytosis, direct membrane fusion, or via receptor-ligand interactions (Caby et al., 2005). Extracellular vesicles are derived from all immune cells and tissue resident cells in the body, playing a role in intercellular communication as well in several different pathological and physiological conditions (Shah et al., 2018, O'Brien et al., 2020).

Recent studies have focused on delineating the molecular profiles, potential functional roles, and specific biological properties of milk-derived extracellular vesicles (**MEV**) and their content across various mammalian species (O'Brien et al., 2020). Milk-derived EV and their cargo, including inflammatory-related microRNAs, can be crucial for early disease detection, as reported by Tzelos et al., (2022), where inflammatory-associated microRNAs were associated with bovine mastitis. In addition, in vitro studies using bovine mammary epithelial cell lines have shown that small MEV isolated from dairy cows conferred protection against heat stress through modulation of target genes related to oxidative stress (Castellani et al., 2025). Importantly, it has been reported that depending on the level, dietary bovine milk-microRNAs packed into EV can partially resist enzymatic digestion in the gut while reaching target tissues (López de las Hazas et al., 2022). Therefore, MEV could regulate several physiological and inflammatory processes via maternal-neonatal communication by transferring a complex mix of bioactive molecular cargo, including microRNAs, proteins, and lipids that modulate immune function, support cellular development, and intestinal homeostasis in the newborn calf. Evidence in the literature indicates that EV in milk are secreted by mammary epithelial cells and by immune cells, and that EV cargo differ upon different conditions, including inflammation (Reinhardt et al., 2013; Tzelos et al., 2022) and stage of lactation (van Herwijnen et al., 2016; Samuel et al., 2017). Within this scenario, bovine colostrum-derived extracellular vesicles (**BCEV**) and their cargo may play crucial roles in cellular growth and in innate responses against pathogenic infections (Samuel et al., 2017). However, characterization of BCEV is limited, which may be attributed to the challenges involving the isolation of relatively pure EV from colostrum.

Currently, there is not a single method that allows a comprehensive characterization of BCEV, including phenotype, size, and fraction concentration of EV. Thus, the Minimal Information for Studies of Extracellular Vesicles (**MISEV**) guidelines were developed by the International Society for Extracellular Vesicles (**ISEV**) with the intent to standardize methods for the isolation, characterization, and functional analysis of EV from different biofluids. Welsh et al. (2024) revealed recent advancements in EV biology, including vesicle heterogeneity, multi-omics integration, and translational potential (Zhang et al., 2024). Overall, EV yield is quantified by methods that allow particle count measurements, which comprise nanoparticle tracking analysis (**NTA**), dynamic light scattering (**DLS**), electron microscopy (**EM**), and single-particle interferometric reflectance sensing (**SP-IRS**), among others (Dragovic et al., 2011; Varga et al., 2014). However, the precise quantification of EV from different samples remains technically challenging due to EV heterogeneity and limitations in current analytical methods (Maas et al., 2015). Milk components that have similar density and size of EV, including milk-fat globules, cellular debris, and casein micelles, present major challenges in the isolation of BCEV (Welsh et al., 2024). Given the emerging role of BCEV in potentially regulating metabolic and inflammatory activities in neonatal calves, this study aimed to evaluate different quantitative and qualitative approaches to assess the morphology, properties, and surface markers of BCEV. The relevance of this study lies in evaluating reproducible complementary approaches to characterize EV in bovine colostrum, despite the lack of standardization for BCEV isolation.

All animal use and procedures for the study were approved by the Institutional Animal Care and Use Committee of Texas Tech University (protocol #2022-1197). Four multiparous Holstein dairy cows were randomly selected from a commercial dairy (Hereford, TX) for colostrum collection. Following routine udder preparation, which included fore-stripping and predipping with a 0.5% iodine-based teat dip and drying the teat ends with a clean cloth towel, all teat ends were scrubbed with an alcohol-soaked gauze pad. The first sample was then aseptically stripped directly into a sterile 50-mL conical centrifuge tube with approximately equal amounts of milk collected from the 4 quarters. Colostrum samples were collected within 4 to 6 h after parturition and immediately frozen at −80°C until BCEV isolation. Before BCEV isolation, samples were skimmed by 4 cycles of centrifugation at 3,400 × *g* for 30 min at 4°C (Centrifuge 5810R, Eppendorf, Hamburg, Germany), with the removal of fat layers between each cycle. The BCEV were isolated from the skim milk samples (n = 4; 30 mL/sample) by ultracentrifugation at 340,000 × *g* for 60 min at 4°C in a fixed rotor (Beckman Coulter 70 Ti fixed-angle rotor, Brea, CA). The EV-enriched fractions (2 mL/sample) were collected and kept at 4°C or −80°C until further analysis. The size and concentration of BCEV were assessed by DLS, which detects size distribution of particles in solution, and by SP-IRIS, which is a nanoparticle sensing approach providing high-resolution particle size distribution profiles and concentrations based on the presence of EV surface markers (particles/mL). Different proteins are expressed in the surface membrane of EV; for instance, CD9 and CD63, members of the tetraspanin family, are among the most widely used molecular markers for the characterization of EV (Andreu and Yáñez-Mó, 2014). Both tetraspanins contribute to the organization of tetraspanin-enriched microdomains that influence vesicle membrane curvature and regulate EV uptake by recipient cells (Duke et al., 2025). Due to their consistent enrichment on EV membranes across cell types and species, CD9 and CD63 are routinely employed as positive markers for EV identification and validation, in accordance with current MISEV guidelines (Welsh et al., 2024). The DLS measurements were performed using 100 μL of isolated BCEV diluted in 900 μL of distilled water in a Zetasizer Nano ZS90 (software 7.10, Nano series, Malvern Panalytical, United Kingdom). For SP-IRIS measurements, purified BCEV (25 μL/sample) were loaded onto Flex Lunis chip s(catalog no. 251-1050, Unchained Labs, Pleasanton, CA) that were conjugated to unlabeled anti-CD9 (1 mg/mL; clone IVA50, species reactivity bovine/human, host mouse, catalog no. NB500-494, Novus Biologicals) and anti-CD63 (1 mg/mL; clone CC25, species reactivity bovine, host/isotype mouse/IgG1, catalog no. MA5-28419, Thermo Fisher Scientific; n = 3 replicates/sample) for SP-IRIS analysis (Leprechaun System, Unchained Labs, Pleasanton, CA) and incubated for 1 h at room temperature. Exosome processing followed manufacturer protocols (Unchained Labs, Pleasanton, CA) and used fluorescently labeled primary monoclonal antibodies anti-CD9 (clone IVA50, conjugate Alexa Fluor 647, species reactivity bovine/human, host/isotype mouse/IgG2, catalog no. NB500-494AF647, Novus Biologicals) and anti-CD63 (1 mg/mL; clone CC25, species reactivity bovine, host/isotype mouse/IgG1, catalog no. MA5-28419, Thermo Fisher Scientific). Before exosome labeling, anti-CD63 was conjugated with Alexa Fluor 555 using commercial antibody labeling kit (Alexa Fluor 555, catalog no. A20187, Invitrogen) following the manufacturer's instructions. Single-particle interferometric reflectance sensing measured particle size by using dual-channel fluorescence imaging to detect CD9 (red) and CD63 (green) via a CMOS (complementary metal-oxide-semiconductor) camera, enabling single-particle identification. Single-particle interferometric reflectance sensing operates by illuminating the sensor surface with monochromatic LED light, enhancing nanoparticle scattering through interferometry. The resulting interferometric patterns, recorded by the CMOS detector, correlate with individual particle size and presence on the chip. The SP-IRIS data processing was performed using the Unchained Labs platform (released January 2024; Leprechaun System Software, Unchained Labs, Pleasanton, CA).

To evaluate the integrity of the BCEV membranes, BCEV visualization was performed using 100 µL of freshly isolated BCEV sample (kept at 4°C) diluted in 1 mL of RNase free water. The BCEV (5 μL/sample) were loaded on glow-discharged carbon-coated formvar copper grids for 1 min, negatively stained with 1% uranyl acetate, and air-dried for 1 h at room temperature. The air-dried grids were imaged in a H-7650 transmission electron microscope (**TEM**; Hitachi High-Technologies Corp., Pleasanton, CA) operated at 120 kV at the Texas Tech University College of Arts and Sciences (Lubbock, TX). In addition to TEM, BCEV were visualized by cryogenic EM (**cryo-EM**) and prepared on Lacey carbon EM grids at the Structural Biology Laboratory at UT Southwestern Medical Center (Dallas, TX). Briefly, these grids underwent a preparation step through glow-discharge in a Pelco EasiGlow system (Ted Pella Inc., Redding, CA). The glow-discharged grids were subsequently placed in a humidity chamber within the instrument (Vitrobot Mark IV, FEI) and maintained at 98% humidity. Next, 3 μL of the freshly isolated BCEV samples (kept at 4°C overnight) was applied to the carbon side of EM grids and then blotted for 4.0 s with a blotting force of 5.0. The grids were then rapidly frozen by immersion in pre-cooled liquid ethane using liquid nitrogen (ThermoScientific Vitrobot systems). Following this, the cryo-EM grids were carefully loaded into the autoloader system. The imaging process was carried out using the Glacios Cryo-TEM system (FEI Talos Glacios transmission electron microscope, Thermo Scientific, Hillsboro, OR). Descriptive statistical analysis of the dataset (e.g., DLS and TEM measurements) was performed in SAS (v. 9.4; SAS Institute Inc.). Values are expressed as the mean ± SEM. Despite its common use for EV isolation by size and density, differential ultracentrifugation presents limitations (Willms et al., 2018). For instance, ultracentrifugation might result in a highly heterogeneous sample that has subpopulations of EV with different sizes (Heinemann et al., 2014). Heterogeneity in EV populations represents a substantive challenge during EV characterization because EV comprise of subtypes that differ in size, biogenesis, membrane composition, and molecular cargo (Zhang et al., 2025). Complexity is further amplified in biological fluids, such as milk, where EV coexist with protein aggregates, lipoproteins, and other nanoparticles of similar dimensions. To overcome this technical challenge, a combination of methods was employed for BCEV characterization including DLS, SP-IRIS, TEM, and cryo-EM analyses. Using DLS, we determined that BCEV measured an average of 127.3 ± 9.5 nm in diameter. However, using SP-IRIS analysis, we determined that BCEV had an average diameter of ∼40 to 45 nm using 2 different EV-associated markers CD9 and CD63 (Figure 1A). Additionally, SP-IRIS analysis of the BCEV showed different concentrations (particles/mL) depending on the individual sample and on the EV-associated marker used (Figure 1B). Using TEM analysis, we determined that BCEV were on average 38.7 nm ± 2 in diameter with a spherical shape (Figure 2). The cryo-EM analysis demonstrated that our isolated BCEV have the standard spherical shape morphology with a lipid bilayer (Figure 3, Figure 3). Emerging evidence suggests that EV in biofluids contain nucleic acids with regulatory immune and metabolic functions, which may serve as biomarkers of various diseases in humans and animals (Willms et al., 2018). However, depending on the microenvironment settings, the physiological state, and the donor and recipient cell types, the number of BCEV released and absorbed might vary greatly (Roerig et al., 2021). Previous studies estimated EV concentration based on total protein measurements; however, this often leads to overestimation of the EV yield caused by the presence of high molecular weight proteins (Koh et al., 2017). Isolation and quantification of EV from bovine milk is particularly challenging due to its complex matrix, which contains abundant proteins, lipids, and casein micelles that tend to co-purify with EV, compromising the yield and accuracy of total particle counts (Ukkola et al., 2022). To address these issues, our study applied sample pretreatment strategies including low speed centrifugation to mediate casein dissociation and promote fat removal, followed by sequential ultracentrifugation to separate the EV fraction from colostrum. In our colostrum samples, there were disparities in the diameter of the isolated BCEV between DLS and TEM analyses (larger BCEV detected by DLS relative to TEM). This can be partially attributed to the fact that DLS measurements are based on intensity-weighted size distributions in which accuracy is limited by the influence of large aggregates and the inability to phenotype EV based on morphological features (Frisken, 2001). In contrast, TEM results provided morphological assessment of the isolated BCEV, which showed a well-defined spherical shape of EV in our colostrum samples. Additionally, the inclusion of negative staining with 1% uranyl acetate might have yielded a superior EV definition and lower background on TEM images (Karapanagioti et al., 2025) resulting in higher accuracy in the size distribution of BCEV. In addition to TEM, the cryo-EM images captured in this study provided a better overview of isolation methods and revealed the bilayer boundaries of the EV membranes, supporting the classification based on size and morphology of the BCEV. Novel approaches such as SP-IRIS provided quantitative data on vesicle concentration, size distribution by interferometry, and surface membrane markers via immunofluorescence technology (Deng et al., 2022). Using SP-IRIS, our findings validated the use of EV antibodies markers for bovine CD9 and CD63, which enhanced the detection of bovine species-specific BCEV. Previous studies have yielded an average measurement of BCEV using NTA of 140 ± 5 nm (Santoro et al., 2023). Although NTA provides total particles/EV concentration, the addition of fluorescence labeling (e.g., the use of specific bovine antibodies) with SP-IRIS technology potentially enhanced our detection of smaller BCEV compared with previous reports. In our BCEV samples, TEM and SP-IRIS demonstrated a comparable size distribution across samples. Hence, high-resolution microscopy analyses along with SP-IRIS contribute to a more comprehensive assessment of isolated EV from bovine colostrum, especially at the single-particle level. Overall, these complementary methods can be applied to future studies that use milk-derived EV to downstream molecular analysis to meet ISEV expectations. Such methods can enhance the detection and characterization of EV in studies that evaluate the delivery of EV bio-cargo from parent to target cells as potential mechanisms of therapy and drug delivery (Zhang et al., 2019). Before further conclusions, however, some caveats must be addressed. In the present study, we did not account for the Holstein cows' performance including parity, dystocia at calving, or colostrum quantity and quality to correlate with the BCEV isolation. Fine-scale variations in size distribution profiles can potentially be affected by genetic background, cell of origin, diet, and the health status of the animal (Mecocci et al., 2022). In addition to size, the EV cargo, including lipids, proteins, and microRNAs, is highly dynamic and depends heavily on several factors previously mentioned, especially the health status and cell of origin (Liu and Wang, 2023). However, current evidence suggests that EV physical properties remain relatively stable across different conditions. For instance, although subclinical mastitis substantially alters the microRNA cargo of milk EV with upregulation of immunoregulatory microRNAs such as bta-miR-223-3p, this inflammatory condition does not appear to significantly affect EV size (Saenz-de-Juano et al., 2022). Similarly, when comparing milk with high versus low somatic cell counts, EVs stability in size and morphological characteristics (i.e., CD9 surface marker) were maintained across different sample types (Saenz-de-Juano et al., 2024). Therefore, the findings from this present study can potentially be applied to other milk-derived EV studies in the context of EV specific markers and size measurements, whereas maternal factors and health status profoundly influence the molecular cargo and functional properties of milk EV. Furthermore, a limitation of this study was the small sample size; the present study was exploratory and not powered to detect small effects. Taking into consideration such limitations, our study showed bovine colostrum samples to be rich in EV with standard EV markers, intact bilayer membranes, and measured within the small EV classification size. To strengthen rigor despite the small sample size and the exploratory nature of the study, EV characterization was performed using complementary technologies that interrogate distinct and nonredundant properties of EV: SP-IRIS enabled particle-by-particle sizing and concentration estimates; TEM offered ultrastructural verification of vesicular morphology; and cryo-EM confirmed EV-like structures with minimal sample preparation. Combining visualization (i.e., TEM, cryo-EM) with quantitative, marker-based detection such as SP-IRIS yields a more robust identification of EV in a heterogeneous biofluid such as colostrum.

**Figure 1:**
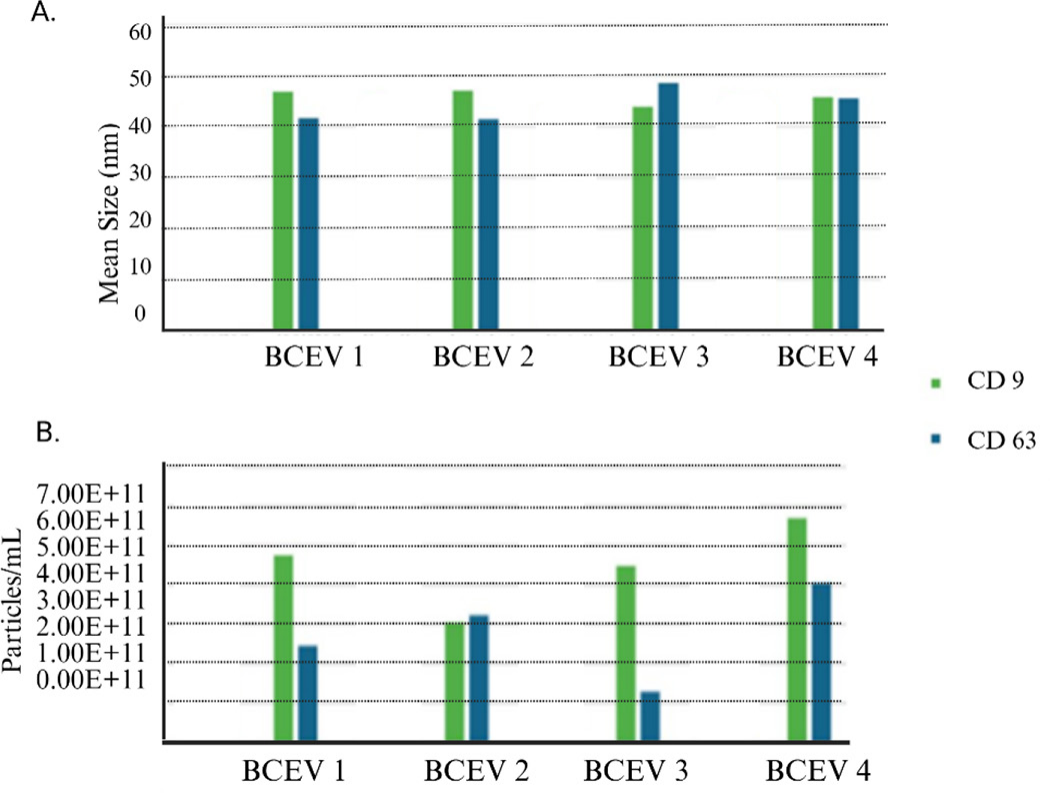
Characterization of bovine colostrum-derived extracellular vesicles (BCEV) by SP-IRIS using EV markers CD9 and CD63. (A) Size distributions for 4 colostrum samples. (B) Particle concentrations (particles/mL) for the same samples. Created with BioRender.com (https://BioRender.com/7wgl8yo).

**Figure 2:**
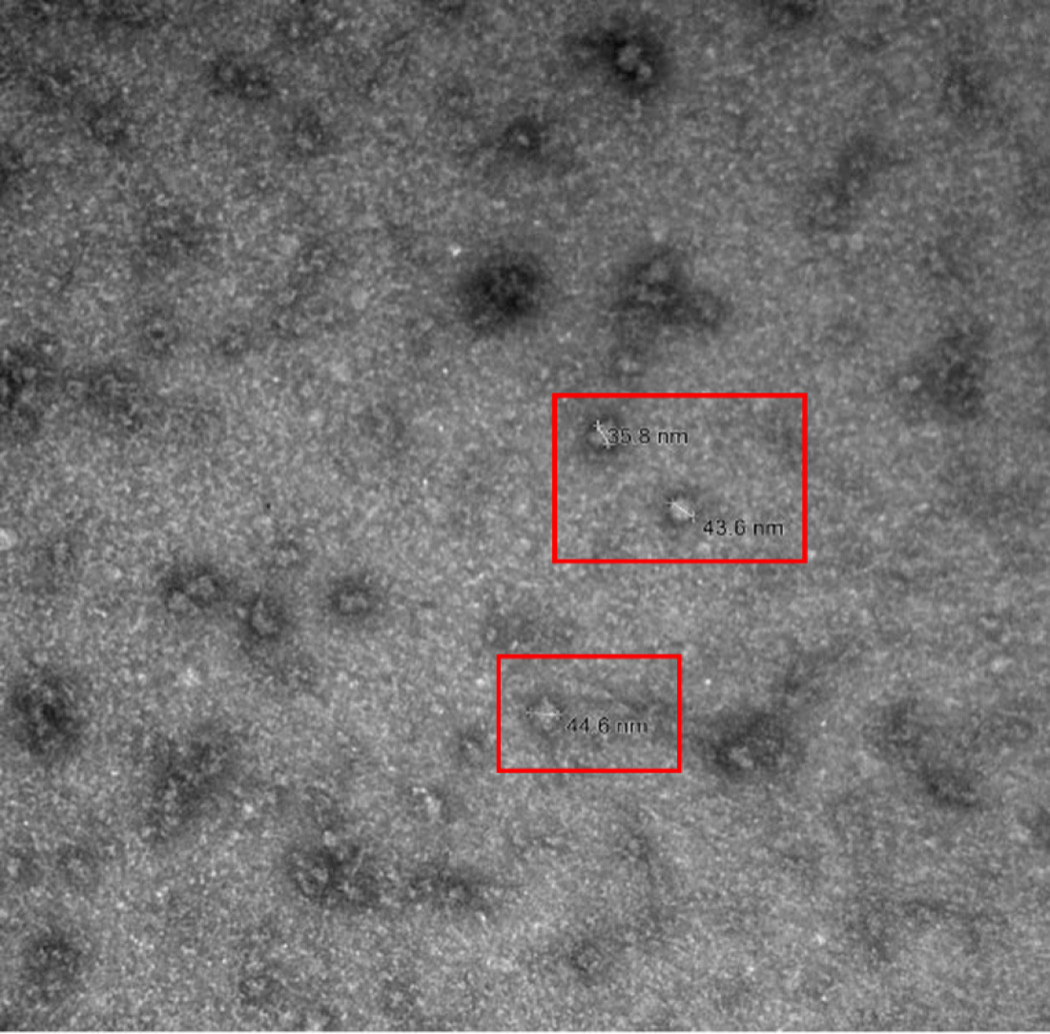
Bovine colostrum-derived extracellular vesicles (BCEV) characterization by transmission electron microscopy. Extracellular vesicles isolated from bovine colostrum were evident, as indicated within the red boxes showing BCEV with well-defined spherical morphology.

**Figure 3:**
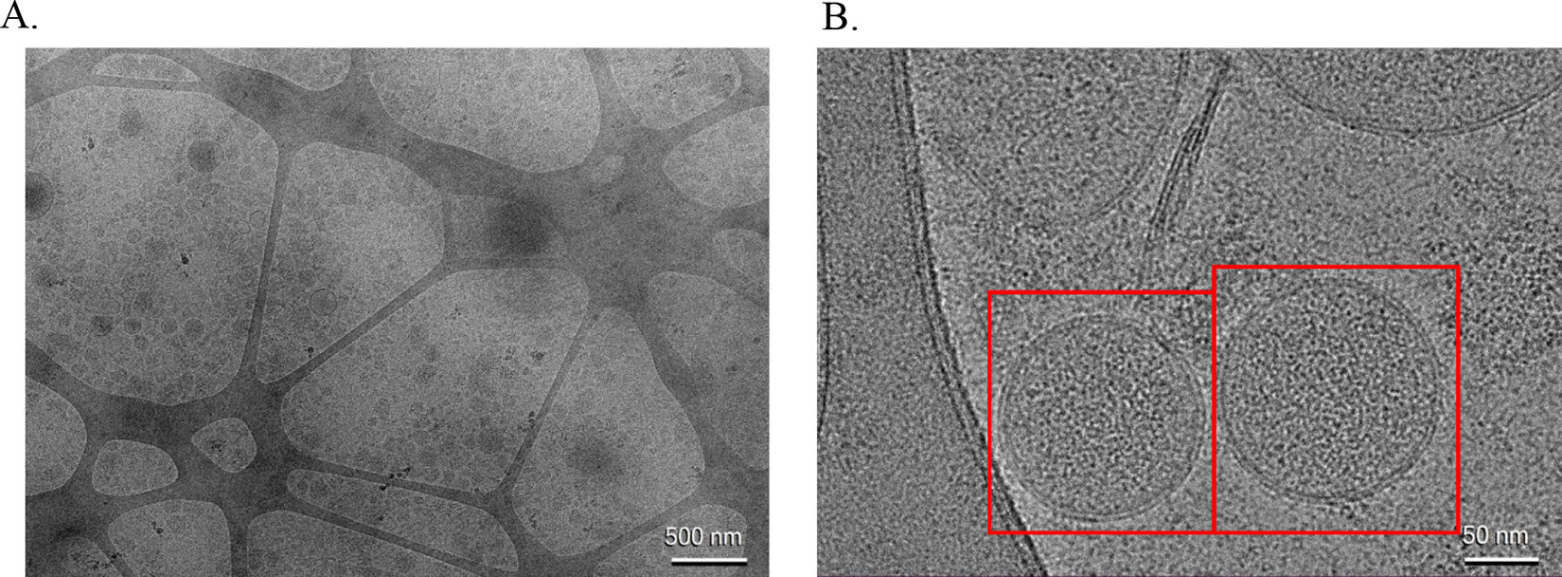
Visualization of bovine colostrum-derived extracellular vesicles (BCEV) by cryogenic electron microscope. (A) Extracellular vesicles isolated from bovine colostrum were detected at 500 nm. (B) Extracellular vesicles were evident, as indicated within the red boxes showing BCEV with well-defined spherical morphology and with extracellular bilayers at 50 nm.
